# Caterpillar Setae–Induced Ocular Complications: A Case Report

**DOI:** 10.1155/crop/4776671

**Published:** 2025-10-12

**Authors:** Nila Kirupaharan, Nicholas N. Fahmy, Marc Estafanous

**Affiliations:** ^1^Department of Ophthalmology, Drexel University College of Medicine, Philadelphia, Pennsylvania, USA; ^2^Department of Ophthalmology, Grinnell College, Grinnell, Iowa, USA; ^3^Department of Ophthalmology, Retina Care Group, Farrell, Pennsylvania, USA

## Abstract

**Purpose:**

This report presents the 2-year follow-up of a rare case of ocular injury caused by caterpillar hairs (setae), highlighting the clinical course, complications, and management strategy, including surgical intervention.

**Methods:**

A 39-year-old male presented 6 months after exposure to a caterpillar cocoon with decreased vision, photophobia, floaters, temporal photopsia, and conjunctival injection. Examination revealed multiple setae embedded in the cornea, conjunctiva, and retina, with associated vitreous inflammation and retinal scarring. The patient underwent pars plana vitrectomy, removal of intraocular setae, and endolaser photocoagulation around retinal entry sites.

**Results:**

The patient's vision gradually improved following surgery, stabilizing at 20/20 visual acuity at 2-year follow-up.

**Conclusion:**

This case highlights a quite rare occurrence of intraretinal caterpillar setae and their potential for severe ocular complications. Prompt recognition, detailed examination, timely surgical intervention, and consistent long-term follow-up are critical to preserving vision. Scleral penetration as a potential mechanism for intraocular setae in similar cases should be considered, emphasizing the importance of maintaining a high index of suspicion and intervening at an appropriate time to minimize the risk of deeper tissue involvement and long-term visual sequelae.

## 1. Introduction

Ocular injuries from contact with caterpillar setae are rare but can result in significant morbidity [1, 2]. The barbed structure of setae facilitates tissue penetration without an exit option, while associated toxins exacerbate inflammation and tissue damage [3, 4]. Most cases involve superficial corneal or conjunctival injuries, with posterior segment complications being infrequent [5]. We present a unique case of intraretinal caterpillar hairs following exposure to a caterpillar cocoon, highlighting the challenges of management and the importance of the right timing of surgical intervention.

## 2. Case Presentation

A 39-year-old male presented to our clinic 6 months after a caterpillar cocoon fell directly into his left eye while he was cleaning in his garage, during which he also rubbed his eyes. Treatment was initiated by the patient's primary care provider, who addressed eyelid swelling and pruritus with topical hydrocortisone 1% cream and artificial tear drops. The patient was referred to a general ophthalmologist for further evaluation. At the time of the injury, there were no signs of infection, such as discharge, fever, or conjunctival chemosis, and no clinical evidence of retained setae or focal granulomas in the eyelid tissue to suggest deeper penetration. At the 6-month presentation, the patient reported decreased vision, photophobia, floaters, temporal photopsia, burning, itching, and conjunctival injection. Visual acuity was 20/50 in the affected eye. These symptoms reflected ongoing ocular irritation consistent with a chronic inflammatory response.

**Figure 1:**
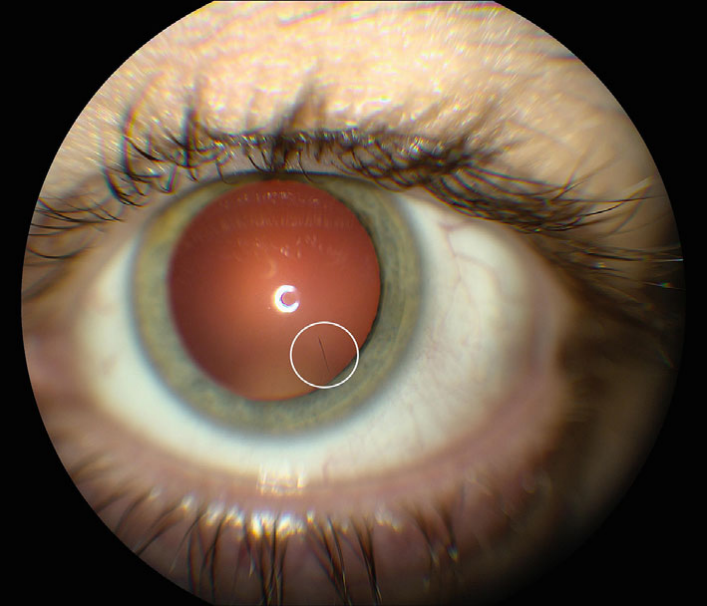
Slit-lamp image showing a caterpillar hair in the vitreous, highlighted within the circled area.

**Figure 2:**
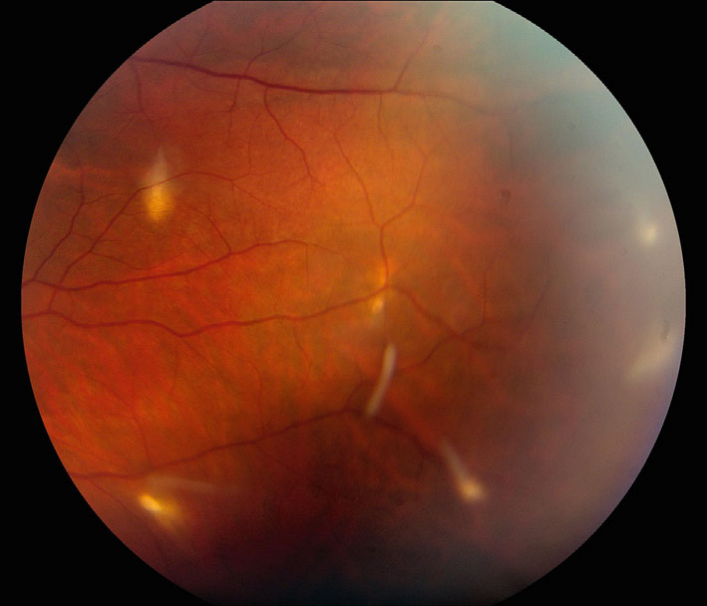
Multiple caterpillar hairs at various stages of retinal penetration, with one hair floating in the vitreous.

Slit lamp examination at that time demonstrated conjunctival hyperemia and revealed multiple caterpillar setae embedded in the cornea and conjunctiva, along with intraretinal setae, trace vitreous cells, and well-circumscribed retinal scars, indicating posterior segment inflammation. There was no evidence of anterior chamber reaction, keratitis, or vitritis to suggest ocular infection. The lens was clear on examination, with no evidence of trauma-induced changes. A superficial corneal hair was removed under topical anesthesia for microscopic examination. Migrating retinal hairs were noted, approaching the vitreous cavity and macula (Figures 1 and 2).

**Figure 3:**
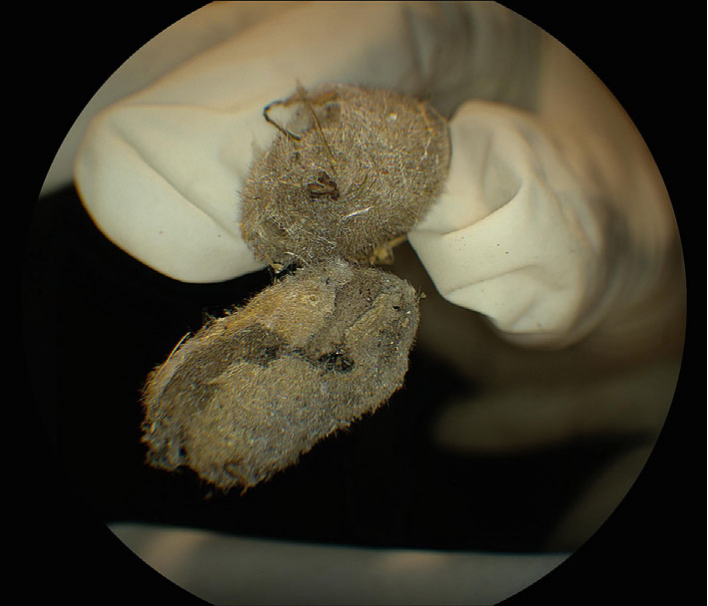
Photograph of a caterpillar specimen with numerous hairs, demonstrating the potential source of ocular injury.

**Figure 4:**
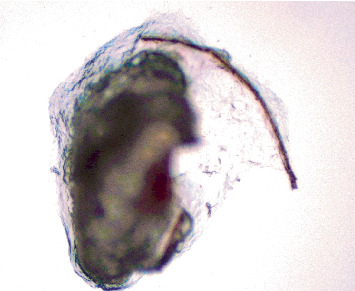
Microscopic picture of caterpillar hair removed from the vitreous cavity.

**Figure 5:**
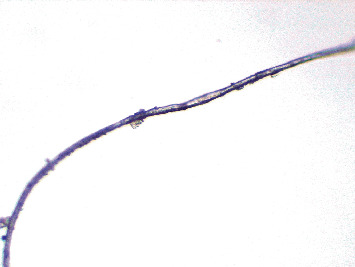
Microscopic picture of caterpillar hair.

The patient underwent pars plana vitrectomy to remove embedded setae from the anterior and posterior segments. Six hairs were excised from the conjunctiva, and four were removed from the vitreous cavity (Figures 3, 4, and 5). Endolaser photocoagulation was applied around the entry points of the setae, resulting in controlled chorioretinal scarring called track-shaped scars (Figure 2). Not all setae were removed; deeper ones within the conjunctiva were left in place, as were portions embedded in the retina. Retinal embedded setae were trimmed with the vitrector when feasible. Induction of a posterior vitreous detachment was avoided due to the patient's young age, firm posterior hyaloid adhesion, and preexisting retinal microbreaks at sites of intraocular hair penetration, all of which increased the risk of retinal tears and detachment.

Postoperatively, the patient was prescribed prednisolone acetate 1% and ciprofloxacin 0.3% ophthalmic drops, each administered four times daily for 1 week, followed by a weekly taper of prednisolone acetate. Postsurgery, encapsulated subconjunctival hairs caused no further irritation, and one retinal hair migrated into the vitreous and remained inert. The retained setae, both subconjunctival and intraretinal, did not elicit further inflammatory responses throughout the follow-up period, likely due to encapsulation or stable positioning. The lens remained unaffected by the surgical intervention and showed no signs of cataract formation or other postoperative complications. At the 2-year follow-up, the patient remained asymptomatic with no new setae in the vitreous cavity and no evidence of continued migration, inflammation, or late-onset complications. His final visual acuity was 20/20.

## 3. Discussion

Caterpillar setae are barbed structures capable of penetrating ocular tissues [6]. Their brittle nature complicates removal, while their toxins, likely water-soluble proteins with esterase, protease, and phospholipase activities, can cause inflammation, liquefaction necrosis, and granuloma formation [7]. Migration within the eye is driven by ocular movements, facilitated by the barbed structure [8].

Cadera et al. classified ocular reactions to caterpillar setae into five types [9]: (1) acute inflammatory response, managed with irrigation, manual removal, and topical antibiotics/steroids; (2) chronic inflammation, which requires removal of residual setae for resolution; (3) nodular conjunctivitis, which may necessitate surgical excision; (4) anterior segment involvement, presenting as iritis and treated with topical steroids; and (5) posterior segment involvement, involving vitreous or retinal penetration and leading to complications such as macular edema or endophthalmitis. Management of Type 5 often requires vitrectomy and corticosteroids.

Our case represents Type 5 involvement, with caterpillar setae embedded in the cornea and conjunctiva, as well as direct scleral penetration, leading to rare intraretinal complications. In our patient, the pattern of wire-straight scleral penetration was evident, with some setae initially visible externally as they pierced the cornea and sclera. Over time, the corneal setae migrated deeper into intraocular structures, and the cornea remained clear. Additional setae were observed within the vitreous cavity, with some embedding into the retina and associated with adjacent chorioretinal scarring. Although Sengupta et al. suggested corneal entry as the primary route for intraocular setae [5], this case demonstrates an alternative mechanism via scleral entry.

Timely surgical intervention was critical to this patient's outcome. The 6-month delay allowed deeper penetration of the setae, increasing the complexity of their removal. However, a combination of vitrectomy, endolaser photocoagulation, and conservative management of embedded setae resulted in excellent visual recovery. Encapsulation of residual setae likely mitigated further inflammatory reactions by isolating the setae from surrounding ocular tissues.

## 4. Conclusion

This case illustrates the potential for caterpillar setae to cause rare and severe ocular complications, including intraretinal involvement. Prompt recognition, thorough medical history, detailed examination, and timely surgical intervention are paramount to preserving vision. Clinicians should maintain a high index of suspicion in cases of ocular exposure to caterpillar cocoons, as delayed treatment can result in deeper tissue penetration and more serious consequences threatening eyesight. Patients with caterpillar injuries will require long-term follow-up at regular intervals to monitor for potential late-onset complications and ensure continued ocular health.

## Data Availability

Data sharing is not applicable to this article as no datasets were generated or analyzed during the current study.
